# Mesenchymal stem cell therapy in amyotrophic lateral sclerosis (ALS) patients: A comprehensive review of disease information and future perspectives

**DOI:** 10.22038/IJBMS.2023.66364.14572

**Published:** 2023

**Authors:** Shahrzad Najafi, Parizad Najafi, Najmeh Kaffash Farkhad, Ghazal Hosseini Torshizi, Reza Assaran Darban, Amir Reza Boroumand, Sajad Sahab-Negah, Mohammad Ali Khodadoust, Jalil Tavakol-Afshari

**Affiliations:** 1 Department of Biology, Mashhad Branch, Islamic Azad University, Mashhad, Iran; 2 Immunology Research Center, Department of Immunology, Faculty of Medicine, Mashhad University of Medical Sciences, Mashhad, Iran; 3 Neuroscience Research Center, Faculty of Medicine, Mashhad University of Medical Sciences, Mashhad, Iran; 4 Shefa Neuroscience Research Center, Khatam Alanbia Hospital, Tehran, Iran

**Keywords:** Amyotrophic lateral sclerosis, Clinical trial, Mesenchymal stem cells, Motor neurons, Neurological disease

## Abstract

Amyotrophic lateral sclerosis (ALS) is a rare deadly progressive neurological disease that primarily affects the upper and lower motor neurons with an annual incidence rate of 0.6 to 3.8 per 100,000 people. Weakening and gradual atrophy of the voluntary muscles are the first signs of the disease onset affecting all aspects of patients’ lives, including eating, speaking, moving, and even breathing. Only 5-10% of patients have a familial type of the disease and show an autosomal dominant pattern, but the cause of the disease is unknown in the remaining 90% of patients (Sporadic ALS). However, in both types of disease, the patient’s survival is 2 to 5 years from the disease onset. Some clinical and molecular biomarkers, magnetic resonance imaging (MRI), blood or urine test, muscle biopsy, and genetic testing are complementary methods for disease diagnosis. Unfortunately, with the exception of Riluzole, the only medically approved drug for the management of this disease, there is still no definitive cure for it. In this regard, the use of mesenchymal stem cells (MSCs) for the treatment or management of the disease has been common in preclinical and clinical studies for many years. MSCs are multipotent cells having immunoregulatory, anti-inflammatory, and differentiation ability that makes them a good candidate for this purpose. This review article aims to discuss multiple aspects of ALS disease and focus on MSCs’ role in disease management based on performed clinical trials.

## Introduction

Amyotrophic lateral sclerosis (ALS) is a rare, deadly, idiopathic neurological disease of the human motor system mainly affecting the neurons responsible for controlling voluntary muscle movements (1). The disorder was first described by Jean-Martin Charcot in 1869 as a special neuromotor disorder (2) and is commonly known as Lou Gehrig’s disease following the retirement of this famous ballplayer in the 1940s due to this disease (3). Because of the progressive nature of ALS, the symptoms become more severe over time (4). In fact, the disease onset is usually very imperceptible but gradually the symptoms progress into palpable weakness or atrophy (5). Early symptoms include twitching muscle involvement (such as arm, leg, shoulder, or tongue), muscle stiffness (spasticity), muscle spasms, and swallowing problems (6, 7). These symptoms are initially focal, but as the disease progresses, they tend to spread throughout the body (5) which usually leads to swallowing difficulty (dysphagia), speech difficulty (dysarthria), and breathing disorder (dyspnea) (8). In this context, respiratory muscle dysfunction is the main cause of death in ALS patients within 3 to 5 years of the symptom’s onset (5). Unfortunately, despite extensive research on ALS, today there is no clinical or prophylactic treatment for it. However, there is only one approved drug called Riluzole to modify the disease in this area (9, 10) with effectiveness of 3 to 6 months in increasing the patient’s mean lifetime. But, this effectiveness varies from person to person (11).

Respecting the above information, finding new treatment strategies can be promising to increase the ALS patient’s life span. In this regard, Mesenchymal stem cells (MSCs) have been used for many years in the treatment of ALS patients in preclinical and clinical studies (12, 13). The migratory behavior, tissue regenerative effect (12, 14), and the ability to differentiate into various cell types like neuron cells (15) has made MSCs suitable candidates in this field. This review article tries to study the role of MSCs in the treatment of ALS in addition to familiarity with different aspects of the disease.

***Factors involved in ALS development***

To date, the cause of ALS is unknown and it remains an unanswered riddle for researchers. However, there is scientific evidence that both genetics and the environment are key players in this scenario (16) as follows:

*Genetics*

Research shows that some particular gene (more than 30 different genes) mutations are associated with motor neuron destruction and ALS development (17). Some of these mutations are directly responsible for the disease induction because they are inherited from parents with infected children and are known as mutated familial ALS genes including SOD1, TARDBP, FUS, OPTN, VCP, UBQLN2, C9ORF72, ANG, SETX, and SQSTM1 (18, 19). 

*Environmental factors*

Many epidemiological studies have examined the environmental factors involved in ALS development (20, 21). Obtained results show that inappropriate lifestyle and various environmental factors may affect the onset and spread of the disease, including exposure to toxins, heavy metals, pesticides, agricultural chemicals, electrical magnetic fields, and viruses (22-24). 

Also, physical activity, physical trauma, diet, smoking, occupational hazards, geographic region, cluster (ALS/Parkinson dementia complex), age, and male gender are considered as other influential environmental factors (25-28). For example, it has been proven that ALS prevalence is higher in men than women (approximately 2 times). 

***Diagnosis***

The ALS diagnosis is mainly based on clinical manifestation showing both upper and lower motor neuron failure and there is no specific single test to diagnose it (29). Also, since the disease can mimic the condition of other neurological diseases, it is difficult and sometimes impossible to diagnose in the early stages (30). Some common tests for distinguishing from other neurological diseases include Nerve Conduction Study (NCS), Magnetic Resonance Imaging (MRI), blood and urine tests, Electromyogram (EMG), muscle biopsy, and Genetic Testing (30, 31). History of notable patient pain or muscle atrophy, physical examination like spirometry test, neuroimaging laboratory, and electrodiagnostic testing could be very helpful in disease diagnosis (32). In addition, diagnostic criteria have been validated and derived from the El Escorial and modified Airlie House criteria (33). Today, another suggestive complementary method for disease diagnosis is the use of clinical and molecular biomarkers (22).

***ALS biomarkers***

The lack of a definitive diagnostic method for ALS, and consequently the rapid progression of the disease due to delayed diagnosis, highlights the need to discover new diagnostic solutions. In this regard, there are various clinical and molecular biomarkers with crucial diagnostic roles (34). 

Some of the most important of them are electromyography (to detect motor neuron damage), transcranial magnetic stimulation (functional integrity of neurons), electrical impedance myography (functional integrity and muscle structure), and neurophysiological approaches (35, 36). Also, molecular biomarkers like inflammatory cytokines are key helpers in this scenario and can be detected in body fluids including CSF, urine, blood, and saliva (35, 37). These biomarkers can be categorized as follows: biomarkers related to excitotoxicity, oxidative stress, inflammation, metabolic dysfunction, neurodegeneration, and other blood biomarkers (37, 38). Some of the most important molecular biomarkers based on previous studies are summarized in Table 1.

***Proposed mechanisms of disease development***

**Figure 1:**
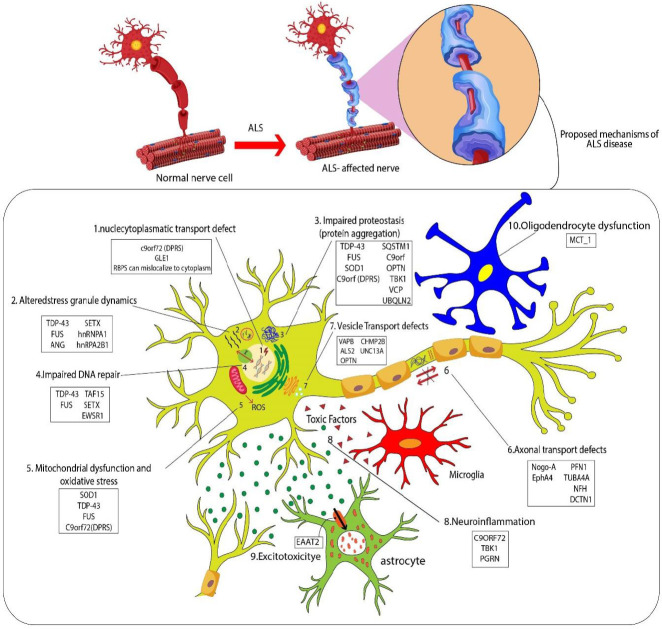
Proposed mechanisms of amyotrophic lateral sclerosis (ALS) disease

Like many other autoimmune diseases, despite global efforts of researchers and clinicians, the causative mechanisms of ALS (particularly in sporadic patients) are still unknown. In fact, several factors and interactions of environment, genetics, age, and other elements are involved in the disease development and progression (56). On the other side, the variety of genetic and phenotypic features between cases has prevented its discovery and general conclusions about the mechanisms of pathogenesis (57). However, some of the most commonly suggested pathogenic mechanisms are: disturbances in RNA metabolism, impaired protein homeostasis, defects in nucleocytoplasmic transport, impaired DNA repair, excitotoxicity, mitochondrial dysfunction, oxidative stress, disturbances in axonal transport, neuroinflammation, oligodendrocyte dysfunction, vesicular transport defects, and alteration in nucleocytoplasmic transport (56, 58) which are schematically given in Figure 1.

***Current treatments strategies***

The complexity of ALS pathology, both from a molecular mechanisms to clinical symptoms, makes it difficult to identify the exact causative factor, and so develop a single drug or targeted treatment (59). On the other hand, unfortunately, despite the increasing prevalence of the disease following the industrialization of most countries in recent decades (which increases the chance of exposure to environmental risk factors) and the efforts of researchers, there is still no definitive cure for ALS (59). However, in most European societies, Riluzole (Sanofi-Aventis, USA) as an anti-glutamatergic drug is used (50 mg twice daily) as the only approved drug in disease management, but it has adverse effects such as liver problems and diarrhea and in the best case can increase the average life expectancy of patients by 3 to 6 months (9, 60). Also, anti-oxidant drugs have been considered by many researchers today due to the crucial role of oxidative stress in ALS induction and/or progression (56). One of the most common anti-oxidant drugs in this field is Edaravone (Mitsubishi Tanabe Pharma Corporation (MTPC), USA), and its safety and effectiveness have recently been studied in several clinical trials (61-63). In this regard, analysis of the results of 2- years treatment of ALS patients with Edaravone on 621 ALS patients (331 patients in intervention- and 290 patients in control groups) showed that the drug is well tolerated by patients but no significant effect on disease progression and respiratory function was reported (64). However, in another study of 22 ALS patients in Korea, the use of Edaravone showed a modest effect on ALSFRS- score and FVC in patients. Also, only minor side effects were reported in this study (63). Although there are still contradictions in the obtained results in this field, today Edaravone as an approved drug to reduce the progression of ALS is accepted in the USA, Japan, Canada, South Korea, and Switzerland (5).

***Other proposed treatment strategies***

In addition to the drug treatments mentioned above, some other treatment methods have also been suggested to control and reduce the disease symptoms, including respiratory support (65), psychological and social support (66), occupational therapy (67), speech therapy (68), and physical therapy (69). Also, using trophic factors, respecting their key role in the motor neurons’ survival and maintenance, has been proposed as potential therapeutic alternatives in ALS disease. Although, unfortunately, the subcutaneous infusion for some of these trophic factors like CNTF and IGF-1 has not resulted in significant therapeutic benefits in clinical trial studies (70), intrathecally injection (IGF-1 and BDNF) has shown moderate improvement results in some cases, without any severe side effects (49), which indicates the need of conducting more animal and human studies in this field.

Genetically modifications like using silencing RNA of the mutant SOD1 or TARDBP, ALS2, and ALS4 genes, in familial ALS patients could be considered as a good optional therapeutic method (71). Design of iron chelating multifunctional molecules like M30 and HLA20, alone or in combination with other compounds, is another valuable approach to promote the motor nerves’ survival via supporting effects on neuro-differentiation and sprouting of axons, leading to reinnervation of muscle fibers (72). Another common method that has been used from the past to the present to treat ALS patients or slow down the disease progression in preclinical and clinical studies is the use of mesenchymal stem cells (which are explained in more detail below) (12, 73).

***Mesenchymal Stem cells (MSCs) and their role in ALS improvement***

**Figure 2.**
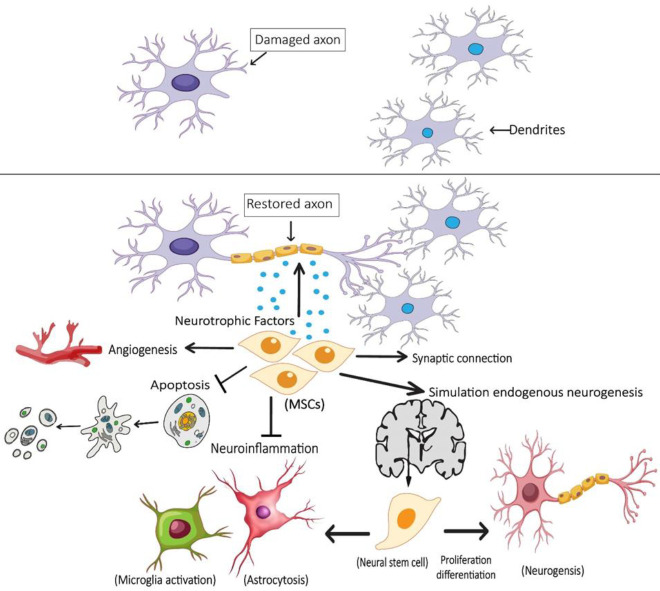
Proposed mechanisms of neurorestoration by mesenchymal stem cells

MSCs are adult stromal multipotent cells first isolated from Bone Marrow (BM) as fibroblastic colony-forming units in 1976 by Friedenstein *et al.* and later detected in many other tissues (74). In the human body, they remain uncommitted until receiving a signal to develop into a specialized cell with new specialized cellular functions (75). In many tissues, they serve as an internal reservoir and are also essential for the growth, development, survival, and repair and construction of various body parts (76). MSCs are found in all multicellular organisms and must exhibit three essential properties based on the International Society of Cellular Therapy (ISCT) guideline. First, proliferation by mitotic cell division to produce progeny is the same as the originating cell (76). Second, MSCs should be able to self-renew over long periods (77). Third, they possess the pluripotent ability to differentiate into different multilineages (e.g., osteocytes, adipocytes, and chondrocytes) under certain physiological conditions (14, 78). They are easily accessible and can be isolated from two cell types: adult sources like BM (79), adipose tissue (80), peripheral blood (81), etc., and fetal sources such as amniotic membrane (82), placenta (83), and umbilical cord (84). To date, these cells and their derivatives have been used in the treatment of various diseases and many clinical trials, including diabetes (85), Covid-19 (86-89), and also neurodegenerative diseases like Alzheimer’s (90), ALS (12), ataxia (91), and Parkinson’s disease (92). These cells have several salient features that make them suitable candidates for the treatment of diseases, including: (1) they do not face ethical considerations associated with the use of Embryonic Stem Cells (ESCs) (93), (2) they can be isolated and expanded both *in vitro* and *in vivo* using a simple method (14, 94), (3) they have multiple immunomodulatory and anti-apoptotic properties through various mechanisms (95, 96), (4) Their limited replication time reduces the possibility of malignant transformation after infusion compared with ESCs and iPSCs (97), (5) they are not immunogenic and do not need immune-suppressive drug consumption before injection due to lack of expression of MHCs given the possibility of autologous transplantation (98), (6) and also they have migratory behavior and can differentiate into multiple cell lines like differentiation of BM-MSCs (15) and chorion-MSCs (99) to functional motor neuron-like cells. The golden role of mesenchymal stem cells in neurological diseases like ALS is due to their role of differentiating into neuronal cells and replacing dead and damaged cells with new functional cells. Also, they help to improve the surrounding environment of neurons by secreting trophic factors and removing toxic molecules, and play a protective role for neurons. (12). Repairing damaged nerve sequences such as dendrites and axons and stimulating alternative brain pathways to improve movement and coordination are other effective mechanisms of these cells in the treatment of ALS patients (98). All these brilliant features make MSCs a good source for cell therapy and regenerative medicine. Figure 2 schematically shows some of the effects of mesenchymal stem cells on neuronal restoration in ALS patients.

***Pre-clinical studies using MSCs in ALS models***

Preclinical research investigating the causes and potential treatments of ALS primarily relies on rat and mouse models, which overexpress mutated human SOD1 genes and exhibit similar patterns of pathology and disease progression to those observed in humans (100). Through the use of these models, researchers have discovered that the transplantation of MSCs via various routes such as intrathecal (IT), intravenous (IV), intramuscular (IM), and intracerebral (IC) can be a safe and effective approach in delaying the decline of motor functions and promoting neurogenesis (101). The secretion of various factors such as cytokines and growth factors like TGF-1 and VEGF is also believed to contribute to the therapeutic protection of neurons and the reduction of inflammation after transplantation (102). Furthermore, studies involving the systematic or intra-spinal administration of MSCs from BM or adipose tissue on standard SOD1 mutant SOD1-G93A mouse/rat have shown significant advantages in terms of delaying degeneration of motor neurons, improving motor function, and extending lifespan (73, 103). A study investigated the impact of combined intra-spinal and systemic injection of MSCs in symptomatic SOD-G93A transgenic rats. The outcomes indicated that MSC grafting had a significant effect on motor activity, grip strength, and lifespan, and led to a greater number of motor neurons that were bigger in size with less apoptosis (104). Another investigation revealed that the intravenous injection of MSCs in SOD1 mice had a notable impact on prolonging survival and reducing symptoms, along with the improvement of multiple histological and biochemical parameters (105). These successful experiments have led to the belief that treating ALS with MSCs could improve neuroprotective or neuro-regenerative properties that modulate biological functions (106). To better understand the significance of these experiments**, **Table 2 provides a summary of the results of preclinical studies conducted in models of ALS with MSCs.

As the results of the above studies show, the use of MSCs in ALS animal models has shown effective results. Delay in motor dysfunction and increase in lifespan are promising results that can be explained by the various mechanisms of action of MSCs, including anti-inflammatory, anti-apoptotic, and immunoregulatory features (110).

***Clinical studies using MSCs in ALS patients***

The first clinical trial that used MSCs to evaluate their safety and potency in the treatment of ALS patients was performed in 2003 by Mazzini *et al*. (111). In this study, autologous bone marrow MSCs dissolved in the patient’s autologous cerebrospinal fluid were injected intrathecally into 7 ALS patients. In terms of safety, these cells were well tolerated in all patients and no serious side effects were observed. Also, MRI images did not show any abnormal structural changes in the spinal cord following cell injection. However, due to the lack of a control group in this study, no results were reported on the effectiveness of the cells, but the good tolerability of cells provided new hope for further research in this area (111). In a 36-month follow-up period, in another study by Mazzini *et al*. in 2006 another promising result was reported. In this study, out of 9 ALS patients participating, 5 patients showed a significant decrease in forced vital capacity (FVC) and ALS- FRS score following cell injection. Also, no serious side effects were observed in any of the participants (112). Taken together, Mazzini *et al*.’s results showed that direct injection of autologous expanded MSCs into the spinal cord of ALS patients is protected without any toxicity and well tolerated by patients (112). In 2012, Mazzini *et al*. reported the results of a 9-year long-term follow-up of 19 ALS patients receiving autologous BM-MSCs. In this study, as expected, no serious side effects related to cell injection like tumor formation were reported in any of the patients. There was also a significant decrease in disease progression and an increase in life expectancy in 6 patients (113). In another exploratory clinical trial new aspects of the immunoregulatory properties of MSCs appeared. Flow cytometry analysis of peripheral blood monocytes of 5 ALS patients showed significant changes in T lymphocyte subtypes following intrathecal and intravenous injection of MSCs. Obtained results showed a 72% increase in T-regulatory subsets (CD4+ CD25+ T cells) and 30–60% decrease in CD86^+^, CD83^+^, HLADR^+^ myeloid dendritic cells, and CD4+ activated cells 24 hr after MSCs transplantation (114). Following the advancement of sciences, the number of clinical trials associated with ALS has increased in recent years. For example, in a recent study conducted by our research team on 15 ALS patients, promising results were observed. In this study, 3 months after simultaneous IV and IT injections of BM-MSCs, a significant increase in ALS-FRS and FVC was observed. Also, no serious side effects were observed in any of the patients (12). Table 3 summarizes the results of some performed clinical trials in this field. 

**Table 1:** Selective molecular biomarkers of amyotrophic lateral sclerosis (ALS)

**Biomarkers**	**Associated process**	**Identification method**	**Finding**	**Ref**
CCR2	Inflammation	chemiluminescent assay & ELISA	Low monocyte expression Low PBMC expression Less CCR2 + PBMCs in limb versus bulbar onset	(37, 39)
IL-4 & IL-6	Inflammation	ELISA	High serum level in hypoxic patients	(40, 41)
MCP-1	Inflammation	RT-PCR	High plasma level	(37, 42)
TNF-α	Inflammation	ELISA	High plasma level	(37, 43)
Caspase-9	Neurodegeneration	ELISA	High serum levels correlated with severity and duration	(37, 44)
NFL	Neurodegeneration	ELISA	High serum level	(37, 42)
PNF-H	Neurodegeneration	ELISA	Plasma and serum level correlated with ALSFRS-R decline	(37, 42)
Cystatin C	Neurodegeneration	Enzyme-linked immunosorbent assay & ELISA	High plasma level	(37, 45, 46)
Nitric Oxide	Excitotoxicity and oxidative stress	Griess nitric colorimetric assay/ELISA	High serum level correlated with duration	(37, 47)
SOD1	Excitotoxicity and oxidative stress	Enzymatic activity assay/ELISA	Low erythrocyte activity correlated with dis ease status	(37, 45, 48)
G6PD	Excitotoxicity and oxidative stress	Enzymatic activity assay/ELISA	Correlated with severity	(37, 49)
Prostaglandin E2	Excitotoxicity and oxidative stress	ELISA	High serum level	(36, 44)
LDL/HDL ratio	Metabolic dysfunction	Not specified	High plasma level correlated with survival	(37, 50)
CNTF	Metabolic dysfunction	ELISA	High serum level	(37, 51)
N-acetyl aspartate	Metabolic dysfunction	MRI & ELISA	High serum level correlated with progression	(37, 51)
Apolipoprotein E	Metabolic dysfunction	PCR & ELISA	Plasma level correlated with progression and survival	(37, 52)
MMP-2	Other blood biomarkers	Sandwich ELISA	Correlated with severity	(37, 53)
MMP-9	Other blood biomarkers	ELISA	High serum level	(37, 53, 54)
TDP-43	Other blood biomarkers	ELISA/ WESTERN BLOT & NMR	Cytoplasmic lymphomonocyte location in ALS subtype	(37, 55)

CCR2: C-C chemokine receptor type 2; IL: interleukin; MCP-1: monocyte chemoattractant protein; TNF- α: tumor necrosis factor α; NFL: neurofilament light chain; pNF-H: phosphorylated neurofilament heavy chain; SOD1: Cu/Zn superoxide dismutase/ G6P; Glucose-6-phosphate dehydrogenase; HDL: high-density lipoprotein; LDL: low-density lipoprotein; CNTF: ciliary neurotrophic factor; MMP: matrix metalloproteinase; TDP-43: TAR DNA binding pro

Nucleocytoplasmatic transport defect, altered stress granule dynamics, impaired proteostasis, impaired DNA repair, mitochondrial dysfunction and oxidative stress, axonal transport defects, vesicle transport defects, Neuroinflammation, excitotoxicity, and oligodendrocyte dysfunction are the main proposed mechanisms of ALS onset in patients. (The figure is created using Adobe Illustrator 2020)

Mesenchymal stem vells (MSCs) secrete various cytokines, and growth factors including neurotrophic factors like transforming growth factor (TGF)-1 and vascular endothelial growth factor (VEGF) contribute to therapeutic protection of neurons, promote endogenous neuronal growth, neurogenesis, and angiogenesis, promote synaptic connection and remyelination of damaged axons, reduce apoptosis, and regulate inflammation mainly through paracrine actions. (Figure is created by Adobe Illustrator 2020)

**Table 2:** Preclinical application of mesenchymal stem cells in amyotrophic lateral sclerosis models

**Rodent model **	**MSC Source**	**Dose **	**Administration**	**Main results**	**Ref**
Irradiated pre-symptomatic male SOD1G93A mice	hBM-MSC	3 × 10^6 ^cells	IV (Tail vein)	Increase in survival Delayed disease onset/progression Delayed the loss of motor function	(73)
SOD1^G93A^ rat	hBM-MSC	3.6×$10^{5}$ cells	IM	Increased lifespan by 18-28 days Preservation of neuromuscular junctions and corresponding motor neurons Delay in motor dysfunction	(103)
Symptomatic SOD1^G93A^ rat	rBM-MSC	2 × 10^6 ^cells	IT	Increased survival Delayed disease onset/progression Delayed the loss of motor function	(107)
SOD1^G93A^ mice	hBM-MSC	1 × 10^6^ cells	IT	Increased lifespan by 8 days Slowed decline in the rotarod test Increased motor neuro survival	(108)
SOD1^G93A^ mice	mBM-MSC	1 × 10^6^ cells	IV	Increased lifespan by 17 days Decreased activated astrocyte and microglial cells Improvement in profile of oxidative stress/ antioxidant enzyme expression	(105)
SOD1^G93A^ mice	hBM-MSC	5 × 10^5^ cells	IT	Increased lifespan by 14 days Delayed disease onset Reduced astrogliosis	(109)

hBM-MSCs: Human bone marrow mesenchymal stem cell; rBM-MSC: Rat bone marrow mesenchymal stem cell; mBM-MSC: Mouse bone marrow mesenchymal stem cell; IV: Intravenous; IM: Intramuscular; MSCs: mesenchymal stem cells; ALS: amyotrophic lateral sclerosis

**Table 3.** Clinical applications of mesenchymal stem cells in amyotrophic lateral sclerosis patients

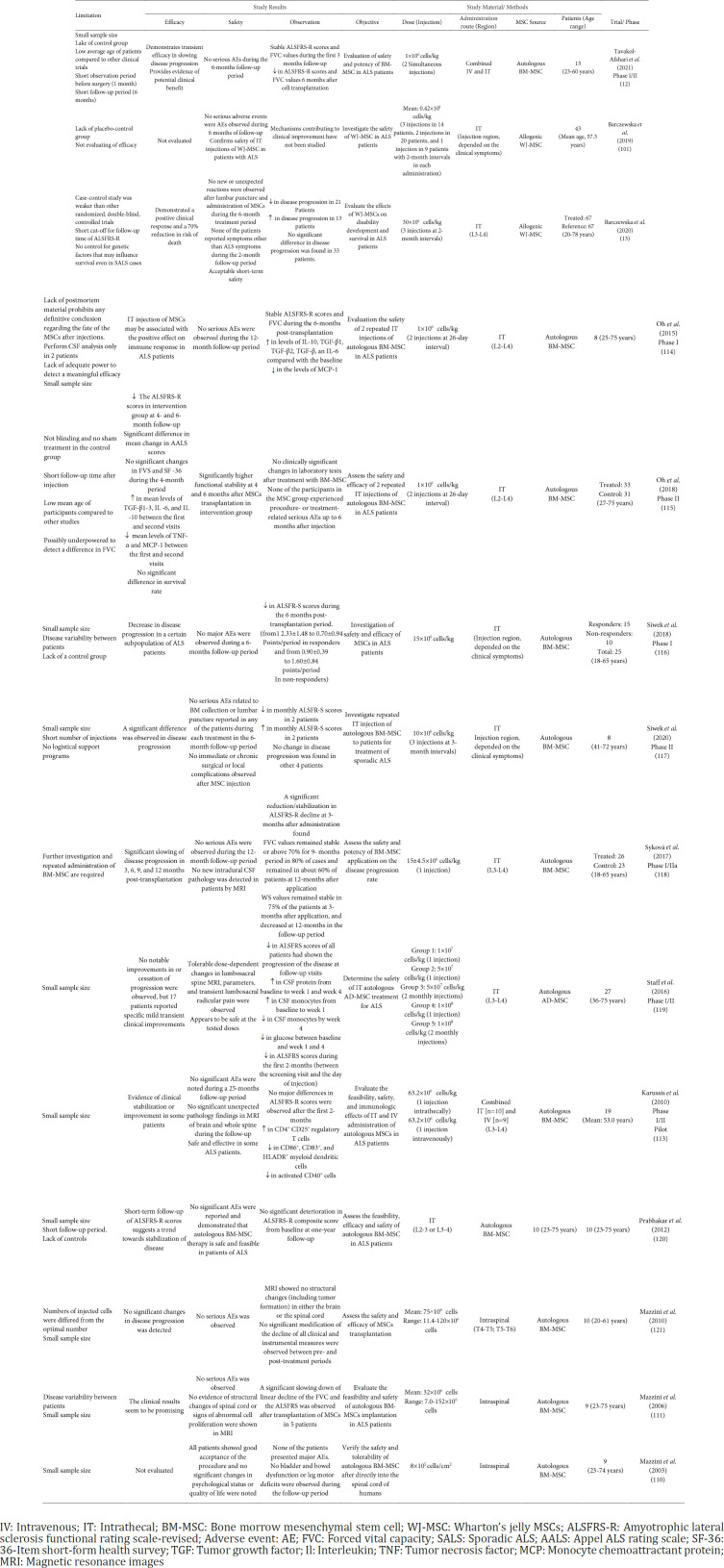

## Conclusion

To sum up, ALS is a fatal neuro-degenerative disease affecting all aspects of the sufferer’s lifestyle including speaking, swallowing, breathing, moving, and their survival. To date, with the exception of Riluzole for disease management, there is no definitive cure for ALS. In this regard, MSCs are considered a good therapeutic approach due to their brilliant features like anti-inflammatory, immunoregulatory, and differentiation ability. There are many pre-clinical and clinical studies using MSCs in ALS management with promising results. This article aimed to collect general information and available data in this field. 

## Authors’ Contributions

S SN had the idea for the article. SH N, P N, N KF, GH H, and MA KH performed the literature search and provided the first draft of the manuscript. SH N, P N and GH H made the first draft of art works. J TA, AR B, R AD, N KF and S SN scientifically updated the literature search and critically revised the whole work including art works. All authors read and commented on the final draft of the manuscript.

## Conflicts of Interest

The authors declare no conflicts of interest.
